# Effects of Mechanosensory Input on the Tracking of Pulsatile Odor Stimuli by Moth Antennal Lobe Neurons

**DOI:** 10.3389/fnins.2021.739730

**Published:** 2021-10-07

**Authors:** Harrison Tuckman, Mainak Patel, Hong Lei

**Affiliations:** ^1^Department of Mathematics, William & Mary, Williamsburg, VA, United States; ^2^School of Life Sciences, Arizona State University, Tempe, AZ, United States

**Keywords:** olfaction, sensory integration, odor pulse, antennal lobe model, SK channel, moth olfactory dynamics, odor plume tracking

## Abstract

Air turbulence ensures that in a natural environment insects tend to encounter odor stimuli in a pulsatile fashion. The frequency and duration of odor pulses varies with distance from the source, and hence successful mid-flight odor tracking requires resolution of spatiotemporal pulse dynamics. This requires both olfactory and mechanosensory input (from wind speed), a form of sensory integration observed within the antennal lobe (AL). In this work, we employ a model of the moth AL to study the effect of mechanosensory input on AL responses to pulsatile stimuli; in particular, we examine the ability of model neurons to: (1) encode the temporal length of a stimulus pulse; (2) resolve the temporal dynamics of a high frequency train of brief stimulus pulses. We find that AL glomeruli receiving olfactory input are adept at encoding the temporal length of a stimulus pulse but less effective at tracking the temporal dynamics of a pulse train, while glomeruli receiving mechanosensory input but little olfactory input can efficiently track the temporal dynamics of high frequency pulse delivery but poorly encode the duration of an individual pulse. Furthermore, we show that stronger intrinsic small-conductance calcium-dependent potassium (SK) currents tend to skew cells toward being better trackers of pulse frequency, while weaker SK currents tend to entail better encoding of the temporal length of individual pulses. We speculate a possible functional division of labor within the AL, wherein, for a particular odor, glomeruli receiving strong olfactory input exhibit prolonged spiking responses that facilitate detailed discrimination of odor features, while glomeruli receiving mechanosensory input (but little olfactory input) serve to resolve the temporal dynamics of brief, pulsatile odor encounters. Finally, we discuss how this hypothesis extends to explaining the functional significance of intraglomerular variability in observed phase II response patterns of AL neurons.

## 1. Introduction

The ability of insects to track and locate an odor source mid-flight is critical for finding food and mates, but this olfactory navigation task is a daunting one. While odors emanating from a source initially emerge in the form of continuously diffusing filaments, turbulent air currents rapidly fold, spin, and fragment these filaments into discontinuous odor strands of differing sizes and concentrations intermixed with clear media. Thus, insects rarely, if ever, are presented with an easily discernable concentration gradient, marking an unambiguous trail to an odor source, to follow; rather, insects, while tracking an odor source, encounter an odor plume comprised of brief, fragmented strands of odor, with odor pulses tending to occur with higher spatial frequency and increasing odor concentrations as the source is approached (Murlis and Jones, 1981; Murlis et al., 1992; Vickers, 2000; Carde and Willis, 2008). Insects have therefore evolved to perform olfactory navigation within the natural context of odor stimuli encountered in pulsatile fashion; indeed, behavioral experiments show that intermittent stimuli are more effective at prompting male moths to exhibit source-seeking behavior than continuous odor plumes (Baker et al., 1985, 1988; Baker, 1989; Kaissling and Kramer, 1990; Kramer, 1992), and that moths employ a source-seeking strategy in which they surge upwind upon encountering odor strands and cast across wind when losing contact with odors (Vickers and Baker, 1994).

While it is crucial for an insect to be able to detect individual encounters with odor strands embedded in a natural plume, it may be equally important to sense the duration, or temporal length, of stimulation (roughly corresponding to the size of an odor strand), for two reasons. On the one hand, the size of an odor strand contains information about distance to the odor source—at closer distances, the cloud of odor molecules is less fragmented by air turbulence (Yee et al., 1995; Connor et al., 2018; Pannunzi and Nowotny, 2019), and so animals are expected to receive relatively longer stimulation (due to increased odor strand size) when near an odor source; it is therefore plausible that animals also use stimulus duration (in addition to the frequency of odor pulses) as a measure to assess distance to the odor source (Celani et al., 2014). On the other hand, lengthier response durations may be important in allowing the animal sufficient time to evaluate a stimulus, as it is likely that longer response durations permit greater extraction of information about the odor. Responding rapidly and briefly at the onset of stimulation is an effective way to register the temporal dynamics of pulsatile encounters with odor strands, but the brevity of such responses may impair assessment of stimulus quality. It is possible that the moth olfactory system employs bimodality—sensory integration of olfactory and mechanosensory input—to resolve this conundrum.

Thus, the natural olfactory landscape for a flying insect consists of fragments of odor strands embedded in a windy medium exhibiting turbulently varying air speed, with robust stimulation of olfactory receptors likely to occur when odor fragments are delivered via packets of high speed air flow. The task of locating an odor source therefore requires integration of chemosensory input (encoding odor identity and concentration) and mechanosensory input (encoding change in wind velocity), since both pieces of information must intermingle in order to simultaneously classify the identity of a particular odor and rapidly resolve the spatiotemporal dynamics of pulsatile odor encounters (Mamiya and Dickinson, 2015). Accordingly, integration of olfactory and mechanical input has been observed in several regions of the nervous system (Jarman, 2002; Sane et al., 2007), including a subtype of trichoid sensilla on male hawkmoth antennae (Lee and Strausfeld, 1990) and sensilla chaetica on honeybee antennae (Whitehead and Larsen, 1976). Unfortunately, the details of such sensory integration—the physiological mechanisms by which these two modalities are interleaved within brain networks, the influence of both modalities on network dynamics, and the dynamical and behavioral consequences that ensue from such co-mingling—are as of yet poorly understood.

A highly promising brain region in which to explore chemosensory and mechanosensory integration is the antennal lobe (AL). The AL consists of excitatory projection neurons (PNs) and inhibitory local neurons (LNs), and is the first brain area to substantially process odor information arriving from olfactory receptor neurons (ORNs) in the sensory periphery. Although the AL has primarily been studied within the domain of odor (and *CO*_2_) detection, there exist data in moths, cockroaches, tadpoles, and mice suggesting that AL neurons (in moths and cockroaches) or olfactory bulb neurons (in tadpoles and mice) are actually bimodal, and exhibit responses to both olfactory and mechanosensory stimuli (Walldow, 1975; Boeckh and Polz, 1983; Kanzaki et al., 1989; Zeiner and Tichy, 1998; Han et al., 2005; Brinkmann and Schild, 2016; Iwata et al., 2017; Baker et al., 2019). While a cohesive picture of the influence of mechanosensory input on AL dynamics has not yet emerged, the AL is, however, an ideal system for the study of sensory integration, as AL architecture is well understood, AL odor response dynamics are well-studied, and AL neurons are easy to measure individually and in aggregate. Additionally, the anatomy and physiology of the AL is analogous to that of the olfactory bulb (OB) in vertebrates (Hildebrand and Shepherd, 1997), and hence may provide insight into chemo- and mechano-sensory integration in a broad range of species.

In prior work (Tuckman et al., 2021), we employ a combined experimental and computational approach to study the AL as a structure that integrates input from multiple sensory modalities, rather than focusing solely on ambient olfactory stimuli. We present experimental evidence showing that, in addition to responding to odors, PNs within the moth AL also respond to mechanosensory signals arising from high-speed air flow across the antennae. Specifically, we show that chemosensory and mechanosensory stimuli induce remarkably differing response dynamics within the AL—olfactory input (in the absence of significant mechanical input, i.e., low wind speed) tends to induce long-lasting PN responses that lack temporal precision, mechanosensory input (high speed non-scented air puffs) leads to brief, temporally precise PN responses, and the two in combination (high speed odor-laden air puffs) leads to an approximate superposition of the two response patterns (i.e., PN responses with a large, temporally precise transient component along with a less intense longer-lasting component). We then develop a biophysically detailed model of the moth AL that captures many salient features of moth AL odor responses reported in the literature, and we employ our model to simulate both olfactory and mechanosensory input, with olfactory input represented as a focal signal delivered to a glomerular subset and mechanosensory input represented as a global signal impinging upon all glomeruli. We show that PN responses within our model closely mimic our experimental observations, and we suggest that a slow inhibitory current from LNs to PNs, coupled with the weak but widespread nature of mechanosensory input (in comparison to olfactory input) and a glomeruli-spanning LN network that widely distributes inhibition throughout the AL, may be largely responsible for the starkly disparate AL dynamics we observe experimentally in response to olfactory vs. mechanosensory signals. We further suggest, using our model, that mechanosensory input may actually somewhat diminish the ability of AL activity to parse and classify a set of ambient environmental odors, putting forth the hypothesis that the role of mechanosensory input may be to help the insect resolve the spatiotemporal structure of environmental odor plumes while possibly sacrificing some accuracy in odor classification in the process.

Our prior modeling work, however, only examines the dynamics of mechanosensory and olfactory responses within the AL in response to “static” stimuli—i.e., solitary and lengthy (1 s) stimuli. As described above, natural odor delivery, particularly during mid-flight tracking of an odor source, consists of rapid encounters with brief, high wind speed odor pulses. In this work, we extend our model to study the AL response to more naturally structured stimuli, and hence we examine the effect of mechanosensory input on the ability of AL activity to resolve the temporal dynamics of brief odor stimuli delivered in a pulsatile fashion.

## 2. Results

The architecture of our model is based on known properties of a typical insect AL network, and in particular the AL of the hawkmoth *Manduca sexta*. In this moth, the axons of 167,000–251,000 ORNs on each antenna (Homberg et al., 1989) ipsilaterally innervate 64–70 glomeruli in the AL (Rospars and Hildebrand, 1992; Grosse-Wilde et al., 2011), suggesting that roughly 64–70 different types of olfactory receptors are distributed among the ORNs. ORN axons synapse onto a total of about 360 LNs and 1,200–1,300 PNs within the AL (Homberg et al., 1988, 1989), with these cells anatomically segregated into glomeruli and each glomerulus tending to receive input from ORNs expressing a common olfactory receptor. PN axons leave the AL in one of five or six antennocerebral tracts (Homberg et al., 1988; Ian et al., 2016). About 10% of PNs are multiglomerular, whereas most are uniglomerular (Homberg et al., 1988). Additionally, an estimate of 80–100 centrifugal neurons provide feedback to the AL (Homberg et al., 1988), at least one of which responds to both olfactory and mechanosensory stimuli (Zhao et al., 2013). Given the complexity of AL circuits and a lack of quantifiable physiological properties of many circuit elements in the AL, we simplify our model to six glomeruli with canonical circuit connections.

We construct a realistic, large-scale, spiking-network model of the moth AL consisting of PNs and LNs organized into six glomeruli, with 10 PNs and 6 LNs per glomerulus (Hildebrand et al., 1979; Matsumoto and Hildebrand, 1981; Kingan and Hildebrand, 1985; Hoskins et al., 1986; Christensen and Hildebrand, 1987; Homberg et al., 1988, 1989). Individual PNs and LNs are governed by integrate-and-fire spiking dynamics, with random but fixed network connectivity—LNs synapse onto other LNs within the same glomerulus and onto PNs both within and across glomeruli, while PNs synapse only onto PNs and LNs within the same glomerulus (Lei et al., 2004; Reisenman et al., 2004). LNs are GABAergic and exert their postsynaptic effects through fast GABA_*A*_ receptors (with several ms kinetics) as well as slower-acting metabotropic GABA receptors acting over ~500–1,000 ms time scales. PNs within the model are cholinergic and act synaptically through fast nicotinic acetylcholine receptors; PNs are also equipped with an intrinsic small-conductance calcium-dependent potassium (SK) current that activates following several PN spikes and serves to curtail further spiking (Mercer and Hildebrand, 2002a,b).

**Figure 1:**
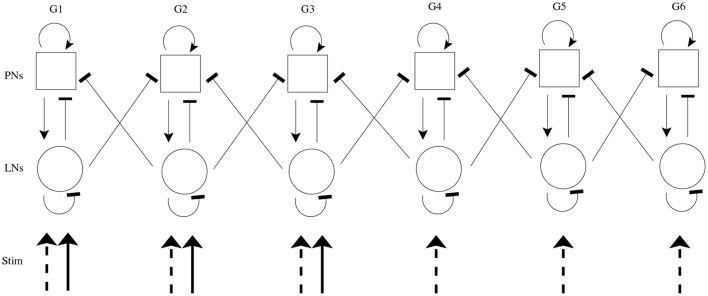
Schematic of model AL network. The model contains 6 glomeruli (columns); squares represent PNs (10 per glomerulus), and circles represent LNs (6 per glomerulus). Arrow heads indicate excitation, while bar heads indicate inhibition. Within a glomerulus, all cell types form synapses with each other (with cell type-specific connection probabilities), while glomerular cross-talk is mediated only via LN→PN synapses (cross-glomerular connection probability is identical for all glomerular pairs). An odor is simulated via delivery of excitatory stimulus current to all cells within a subset of three glomeruli (solid incoming arrows), while mechanosensory input is simulated via delivery of excitatory stimulus current to all cells within all glomeruli (dashed incoming arrows).

We simulate both chemosensory and mechanosensory input to the model, both in isolation and in conjunction. In accordance with the well-established combinatorial odor code employed by olfactory receptor neurons (ORNs) (Joerges et al., 1997; Vickers and Christensen, 1998; Vickers et al., 1998; Malnic et al., 1999; Ng et al., 2002; Wang et al., 2003; Ache and Young, 2005), an odor (in the absence of significant mechanosensory input, i.e., low wind speed) is simulated by delivering an excitatory stimulus current to all cells within a subset of model glomeruli (half of model glomeruli are designated to receive stimulus current); odor identity is equated with the glomerular subset receiving stimulus current. Mechanosensory input (in the absence of odor input) is modeled by sending stimulus current to all cells within the entire AL network. While our prior work is suggestive that PN responses to mechanosensory input tend to be more global than responses to an odor, this cannot be definitively concluded from our prior data (Tuckman et al., 2021), and other work suggests that not all PNs respond to mechanosensory input (Kanzaki et al., 1989); however, we note that the results presented in this paper do not depend on the global nature of mechanosensory input—the model simply requires mechanosensory input to be somewhat more widespread (which is biologically reasonable if ORNs deliver mechanosensory input) and weaker than odor input. If both of these features exist, then activation of the globally connected LN network allows rapid suppression of relatively weak PN responses, ensuring transient responses to mechanosensory input. In order to maintain similar net levels of excitation to the AL in the case of an olfactory vs. a mechanosensory stimulus, the strength of the stimulus current in the mechanosensory case is reduced by a factor of $\frac{1}{2}$ relative to stimulated glomeruli in the olfactory case; this facilitates direct comparison of network dynamics in response to a global signal (mechanosensory input) vs. a focal signal (odor input). Figure 1 shows a schematic of the model network.

Thus, we employ three stimulus scenarios: (i) odor only, with no mechanosensory input, which simulates odor delivered at low wind speed; (ii) mechanosensory input only, with no odor input, which simulates a non-scented, high speed air puff; (iii) additive sensory integration, simulating high wind speed odor delivery, enacted via simple summation of the current pulses in the odor only and mechanosensory only scenarios. We note that in our prior work (Tuckman et al., 2021) we also employed another distinct paradigm of high wind speed odor delivery (the normalized paradigm), simulated by halving then summing the current pulses in the odor only and mechanosensory only scenarios. However, we found that the additive paradigm described in (iv) yielded moth AL dynamics in response to high wind speed odor presentation that were more consistent with our experimental data, and hence in this work we do not include the fourth paradigm. Model details can be found in the section 4. We also note that there is no “odor only” scenario in nature, but we explore this scenario as a means to isolate the effects of the two sensory modalities on the system.

**Figure 2:**
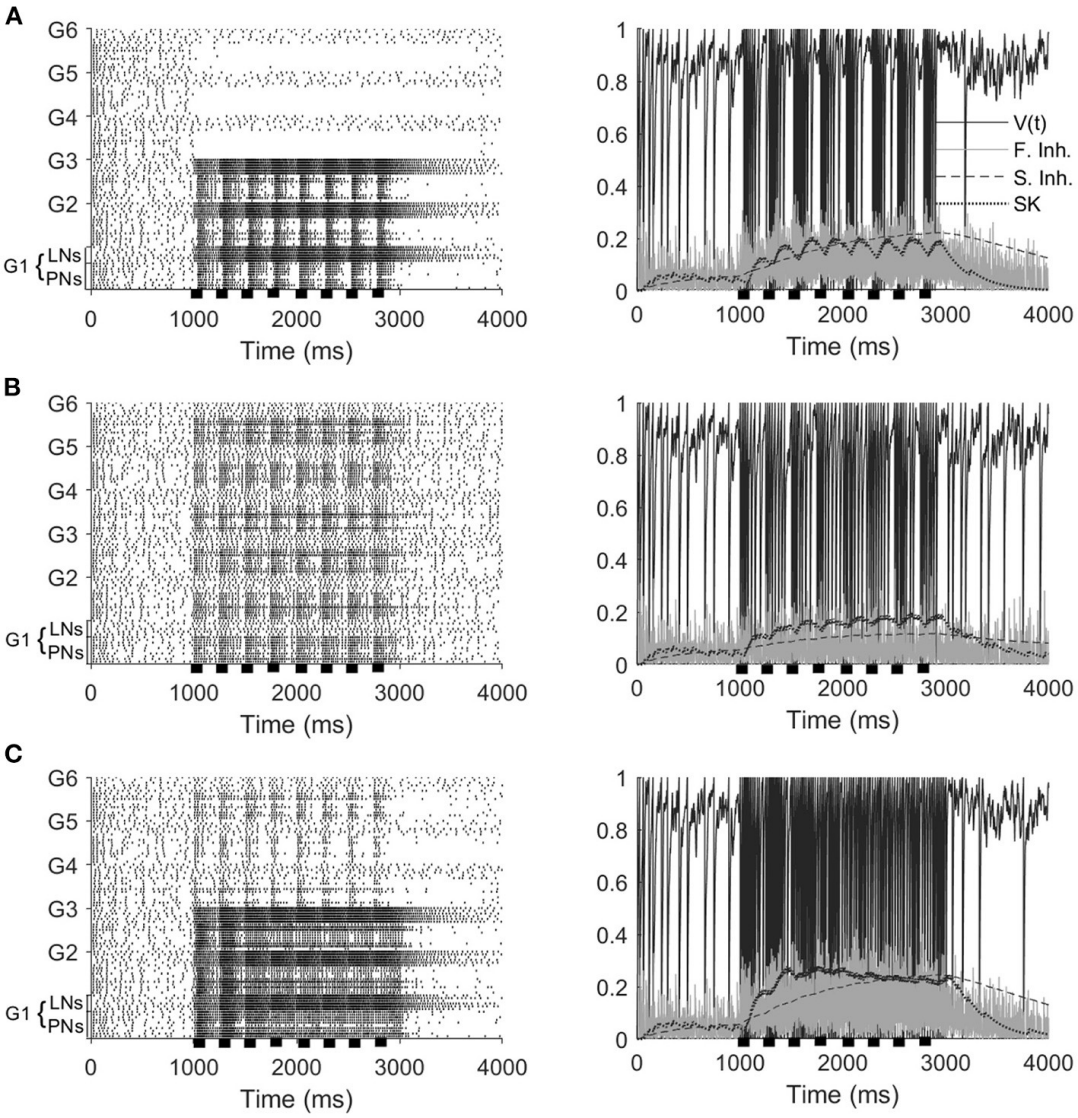
Model responses to 4 Hz stimulus pulses in the odor only **(A)**, mechanosensory only **(B)**, and additive sensory integration **(C)** stimulus scenarios. Odor is simulated by sending stimulus current to only glomeruli 1, 2, and 3, while mechansensory input is simulated by sending stimulus current to all glomeruli. Left panels show spike rasters for the entire network, while right panels show the membrane potential, fast inhibitory current, slow inhibitory current, and SK current for a representative PN within the network (in the presence of odor stimulus, the PN is taken from a glomerulus receiving odor input). Stimulus pulses are 50 ms in length. Black bars indicate stimulus.

Experiments show that a series of short odor pulses evokes a sequence of corresponding spike trains in activated PNs; each individual pulse produces an I_1_ hyperpolarization followed by phase II depolarization (on top of which is superimposed a burst of spikes), with pulse offset eliciting abrupt truncation of spiking activity. The prolonged after-hyperpolarization (AHP) phase, however, does not fully manifest until the end of the final pulse in the stimulus train (Christensen et al., 1998; Lei et al., 2002). Our model PNs, in the stimulus scenarios that include odor, exhibit similar behavior (Figures 2A,C). Within our model, the onset of each pulse initially elicits a brief, synchronous burst of LN spikes that leads to a brief hyperpolarization of PNs (I_1_), with PN spiking ensuing once LNs desynchronize and excitation to PNs ramps up (phase II). Pulse offset yields diminishment of PN spiking as excitation ramps down—LN spiking, on the other hand, does not diminish as rapidly between pulses, and hence the slow inhibitory current to PNs continues to climb during interpulse intervals. At the end of the final pulse in the stimulus train, stimulus-induced excitation to PNs and LNs rapidly vanishes and PN and LN spiking diminishes; however, the slow inhibitory current to PNs, due to its prolonged dynamics, decays over a longer time scale than the stimulus-induced excitation, leading to a lengthy (~1 s) period of PN hyperpolarization as the slow inhibition gradually vanishes (the AHP phase). We also note that within our model PN responses to mechanosensory stimulation alone tend to be briefer and less intense than responses to odor stimulation or combined odor and mechanosensory stimulation (Tuckman et al., 2021).

Furthermore, Figure 2 shows that, at a stimulus pulse frequency of 4 Hz, network PNs receiving stimulus current in the odor only (Figure 2A) and mechanosensory only (Figure 2B) stimulus scenarios appear capable of tracking the temporal structure of pulsatile stimulus encounters. Interestingly, in the additive sensory integration scenario (Figure 2C), PNs that receive both odor and mechanosensory stimulation appear to be less capable of tracking pulse frequency than PNs receiving mechanosensory stimulation alone. This latter result is of particular importance to the present work, as additive sensory integration is the scenario simulating high wind speed odor delivery and hence likely to be most relevant to tracking odor sources. The role played by mechanosensory vs. odor input in the ability of PNs to track the length and frequency of stimulus pulses, as well as the underlying network dynamics, will be explored in the remainder of this paper.

### 2.1. Pulse Length Tracking

We now examine the ability of the spiking response of PNs within our network to discern the duration, or temporal length, of a single stimulus pulse. In our model, an odor is simulated by strongly stimulating a subset of glomeruli, leaving other glomeruli only weakly activated by the concurrent mechanosensory input (which is delivered to all glomeruli). A diversity of glomerular response patterns may therefore emerge, driven by the combination of heterogeneous input glomeruli across glomeruli as well as network interactions among glomeruli.

#### 2.1.1. Stimulus Dependence of Length Tracking

**Figure 3:**
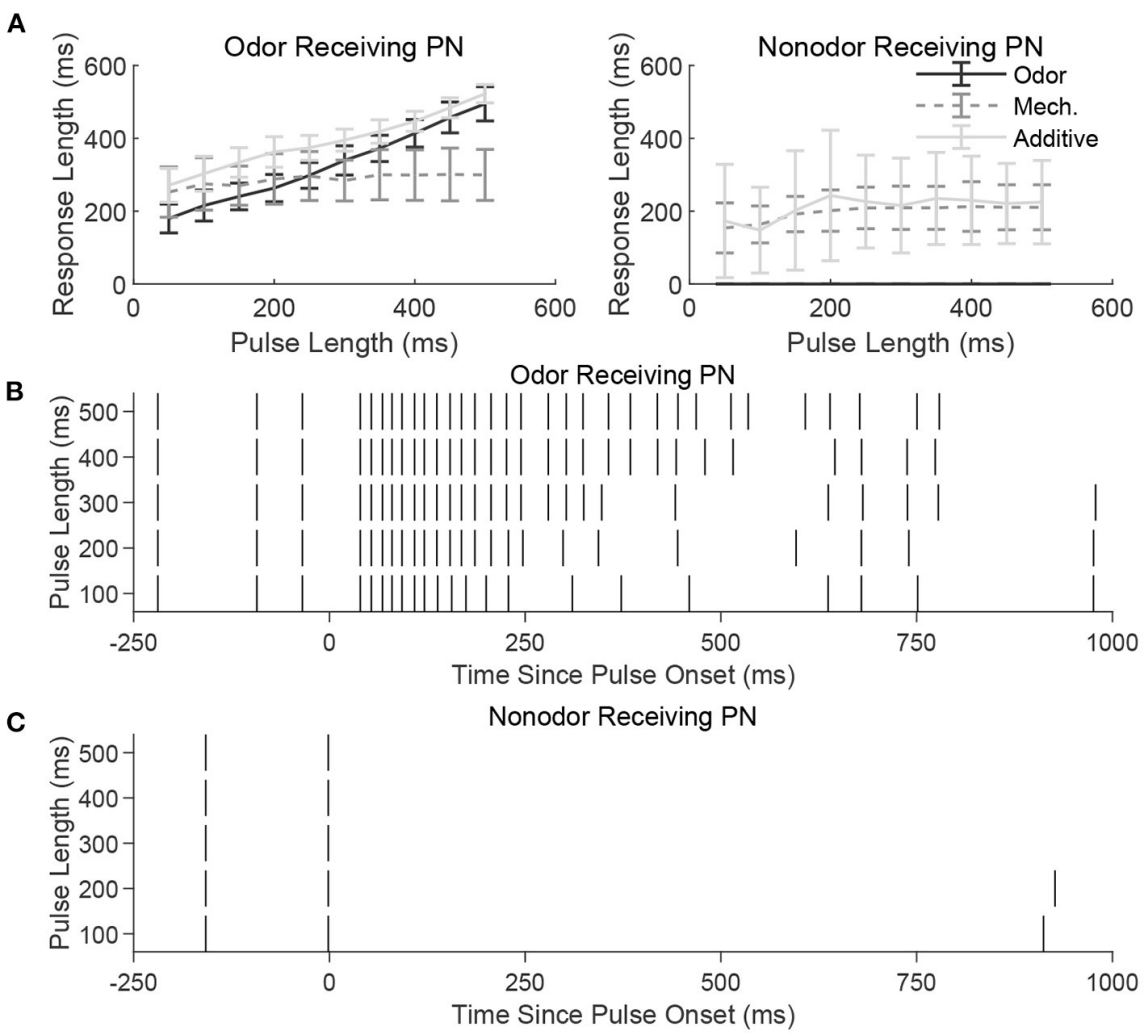
**(A)** Length of spiking response vs. stimulus pulse length for an odor receiving PN (left) and a nonodor receiving PN (right) in the three stimulus scenarios (odor only, mechanosensory only, and additive sensory integration). Responses are averaged over 50 trials. Note that in the presence of odor (odor only and additive scenarios), the odor-receiving PN has a response length that reflects the length of the incoming stimulus pulse, whereas a PN that does not receive odor has a relatively fixed response length regardless of stimulus pulse length. To exemplify this further, we illustrate the spike raster for a single odor-receiving **(B)** and non odor-receiving **(C)** PN at various pulse lengths during the odor only stimulus scenario. The odor-receiving PN has a response that reflects the length of the incoming pulse, whereas the non odor-receiving PN is unstimulated and does not respond. See section 4 for details.

Figure 3A shows the temporal length of the spiking response of a sample model PN as a function of the temporal length of a stimulus pulse, for a PN within an odor-receiving glomerulus (left) or non odor-receiving glomerulus (right). For an odor-receiving PN (Figure 3A, left), within the odor only and additive stimulus scenarios response length increases approximately linearly with pulse length (indicating that the PN response is capable of reflecting and monitoring pulse length), while within the mechansensory only scenario the response curve is relatively flat (suggesting that mechanosensory input alone is not strong enough to enable the PN response to encode pulse length). The reason for this is the interaction between potent widespread inhibition from the glomeruli-spanning LN network and the strength of stimulus-induced excitation. For an odor-receiving PN, stimulus-induced excitation is relatively strong in both the odor only and additive scenarios (due to the potency of odor-induced input), and hence globally pervasive slow inhibition from the LN network is insufficient to quiet these PNs, leading to spiking responses that endure approximately for the length of the stimulus pulse (see Figure 3B for a sample odor-receiving PN in the odor only scenario); in the mechanosensory only scenario, without strong odor input slow inhibition rapidly overpowers any initial spiking response, preventing the neuron from tracking pulse length.

For a non odor-receiving PN (Figure 3A, right), stimulus-induced excitation is relatively weak, as there is no odor-induced input in any of the three stimulus scenarios. The only input is weak mechansensory input (except in the scenario labeled as “odor only,” in which case there is no input at all), and mechansensory input alone does not allow the PN response to track pulse length. Mechanistically, after a potential brief burst of PN spikes at pulse onset, slow inhibition from LNs rises and becomes potent enough to suppress PN responses, leading to brief PN responses that change little with stimulus pulse length (Figure 3C).

#### 2.1.2. Conductance Dependence of Length Tracking

**Figure 4:**
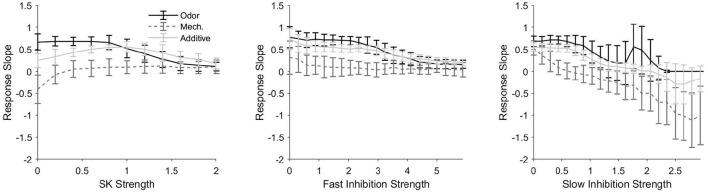
Response slope, defined as the slope of the best fit line for a response length vs. pulse length plot (i.e., plots as in Figure 3A), as a function of the strength of the SK current (left), fast inhibitory synapses (center), and slow inhibitory synapses (right) within the model. A larger (more positive) response slope indicates a greater sensitivity to pulse length, or, in other words, a greater ability of the temporal length of the spiking response to be indicative of the temporal length of the stimulus pulse. In the odor only and additive stimulus scenarios, the PN is taken from an odor-receiving glomerulus; data are shown for a single sample PN and averaged over 50 trials. Values on the x-axes are normalized by the standard strength employed in the model (i.e., 1 represents the standard strength employed for each current in the model).

Considering the apparent variability in the ability of PNs to track pulse length, we now examine the effects of various conductance components—the SK channel-induced current, GABA_*A*_-like fast inhibition, and GABA_*B*_-like slow inhibition—on the ability of model PNs to track stimulus pulse length (Figure 4). We employ a “response slope” metric (the slope of the best fit line for response length vs. pulse length plots, plots such as in Figure 3A) to measure the sensitivity of PN responses to stimulus pulse length. Thus, larger response slopes—i.e., slopes close to one—indicate a greater ability of a PN to monitor pulse length, while response slopes near zero indicate that a PN response does not contain information pertaining to pulse length. We compare how each conductance component affects PN responses in all three stimulus scenarios.

Figure 4 (left) shows the effect of SK current strength on response slope. In the mechanosensory only scenario, the response slope remains near zero for most values of SK current strength, indicating (consistent with Figure 3A) that mechanosensory input alone is insufficient to allow PNs to monitor pulse length; however, for very low values of SK current strength, we find that the response slope is actually negative, indicating that, for these SK strength values, response length tends to *decrease* with increasing pulse length. This latter result arises because the rise time of mechanosensory input is slow for LNs but fast for PNs (this feature is included to prevent an initial LN burst from completely suppressing PN responses to the weak mechansensory input). Consequently, the slow inhibitory current to a PN rises particularly slowly during a pulse of isolated mechanosensory input as LN activity gradually ramps up. As pulse length is increased, there is a greater gradual build-up of slow inhibition to a PN during the pulse, and after pulse offset, the slow inhibition to a PN decays gradually. Thus, in the absence of the SK current, for short stimulus pulses, a PN is capable of responding during the length of the pulse and beyond (since slow inhibition does not build sufficiently during the pulse to suppress PN output during the stimulus decay phase), while as pulse length increases, slow inhibition builds to higher levels during the pulse and curbs PN spiking more rapidly after pulse offset during the stimulus decay phase. In the presence of significant SK current, an auto-inhibitory current that builds more rapidly than the synaptic slow inhibition, the combined potency of the intrinsic SK current and synaptic slow inhibition curtails PN spiking prior to pulse offset for even relatively short stimulus pulses.

In the odor only stimulus scenario, the response slope in Figure 4 (left) tends to decline monotically from near one to near zero as a function of SK current strength, indicating that as the strength of the SK current increases, the ability of the response of an odor-receiving PN to track stimulus pulse length diminishes. This is due to the inhibitory effect of the SK current—the relatively strong odor-induced stimulus current (with modest SK current strength) allows a PN to spike throughout the duration of a pulse (and briefly beyond), while a very strong SK current (acting in conjunction with the gradually building synaptic slow inhibition) tends to curb PN spiking prior to pulse offset.

For the additive sensory integration stimulus scenario in Figure 4 (left), the response slope curve as a function of SK strength appears to be approximately the sum of the curves in the mechanosensory only and odor only scenarios, leading to a nonmonotonic curve with a peak (i.e., a moderate SK strength value at which the response slope, or the ability of a PN response to track pulse length, is maximized). In this scenario, both of the above effects contribute—stimulus-induced input to a PN is very large (mechanosensory plus odor input), and hence for low SK strengths PN responses tend to be substantially prolonged beyond pulse offset (yielding poor tracking of pulse length), while for high SK strengths PN responses tend to be curtailed prior to pulse offset (again yielding poor tracking of pulse length); thus, the ability of a PN response to track pulse length is maximized within a range of moderate SK strength values.

Figure 4 (center) shows that response slope tends to decrease with increases in the strength of fast inhibitory synapses. This occurs because fast inhibition to a PN tends to equilibrate quickly (due to its rapid several ms time course) rather than build gradually; for low to moderate fast inhibition strength values, fast inhibition has little impact on PN firing rate and hence little effect on the ability of a PN response to track pulse length, while very strong fast inhibition dampens and eventually suppresses PN spiking over a relatively fast time scale, yielding brief PN responses that truncate prior to pulse offset (leading to a poor ability to monitor pulse length).

Slow inhibition exhibits a similar trend (Figure 4, right), in that response slope tends to diminish as the strength of slow inhibitory synapses is augmented. As the strength of slow inhibition is increased, the peak magnitude of this gradually building current eventually becomes sufficient to curtail PN responses prior to pulse offset (for sufficiently long pulses); for large slow inhibition strength values, shorter pulses (in which slow inhibition does not have sufficient time to build substantially) allow PN responses to outlast pulse duration, while longer pulses allow sufficient build-up of slow inhibition to curb PN responses prior to pulse offset, yielding potentially negative response slopes.

### 2.2. Pulse Frequency Tracking

We now study the ability of PN responses within our model to track the temporal structure of intermittent stimuli—i.e., to track the frequency of stimuli delivered in the form of a train of pulses. Intracellular recordings from AL neurons using 50 ms odor pulses show that moth PNs act as low-pass filters of pulse rate (each cell tracks odor pulses with bursts of spikes up to a certain cutoff frequency that varies across PNs). Remarkably, PNs have been found that are capable of tracking up to ten odor pulses per second, while pulse rates exceeding a cell's cutoff frequency elicit responses consisting of tonic firing (Christensen and Hildebrand, 1997; Heinbockel et al., 1999). Similar to the previous section in which we analyze the ability of PN responses to track the length of a single stimulus pulse, here we examine how different stimulus scenarios and conductance components affect the ability of PN responses to follow the temporal dynamics of a train of brief stimulus pulses.

#### 2.2.1. Stimulus Dependence of Frequency Tracking

**Figure 5:**
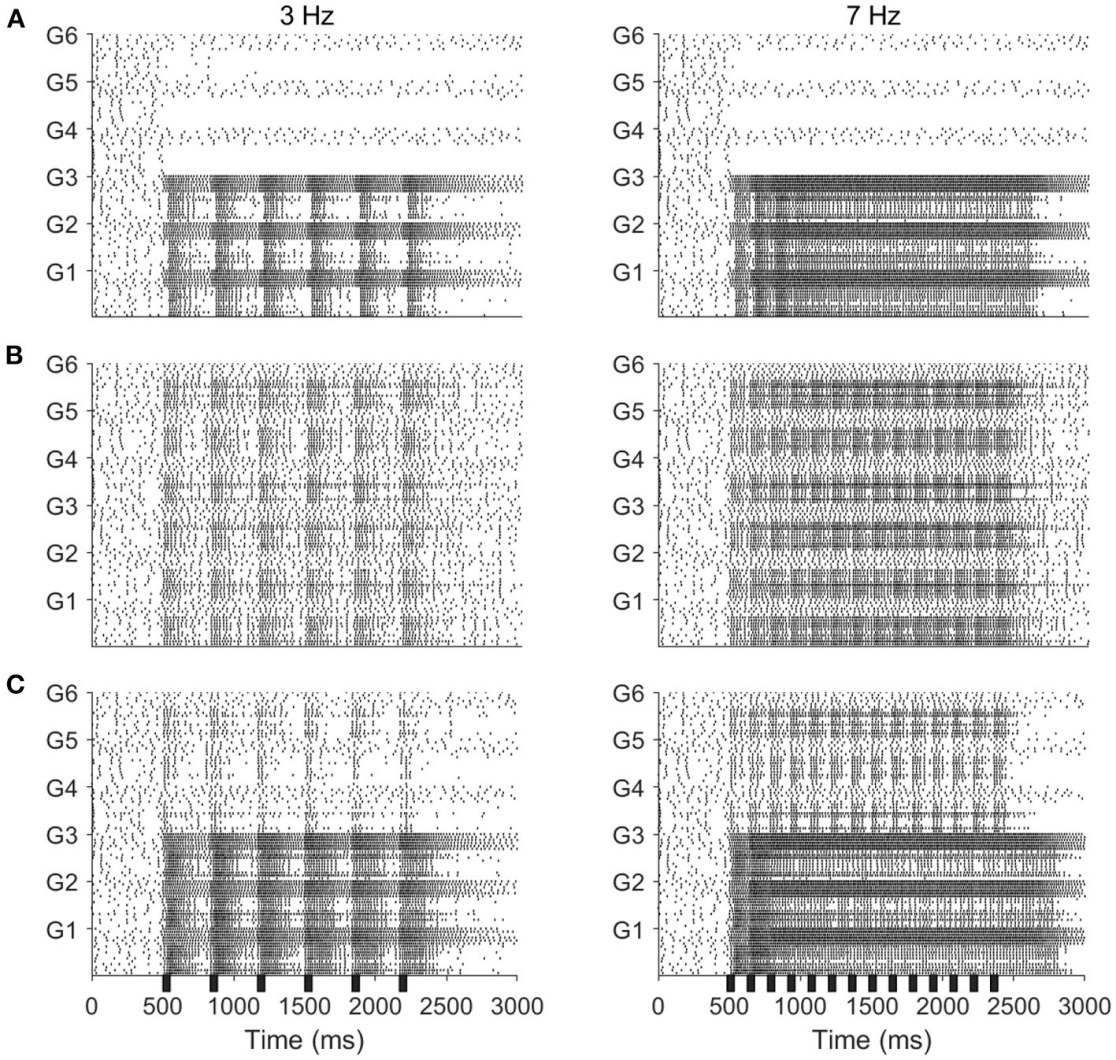
Spike rasters of the model network for 50 ms stimulus pulses delivered at a frequency of 3 Hz (left) or 7 Hz (right), in the odor only **(A)**, mechanosensory only **(B)**, and additive sensory integration **(C)** stimulus scenarios. Odor is simulated by sending stimulus current to only cells within glomeruli 1, 2, and 3, while mechansensory input is simulated by sending stimulus current to cells within all glomeruli. Within each glomerulus, the bottom 10 cells are PNs while the top six cells are LNs. Black bar represents stimulus.

**Figure 6:**
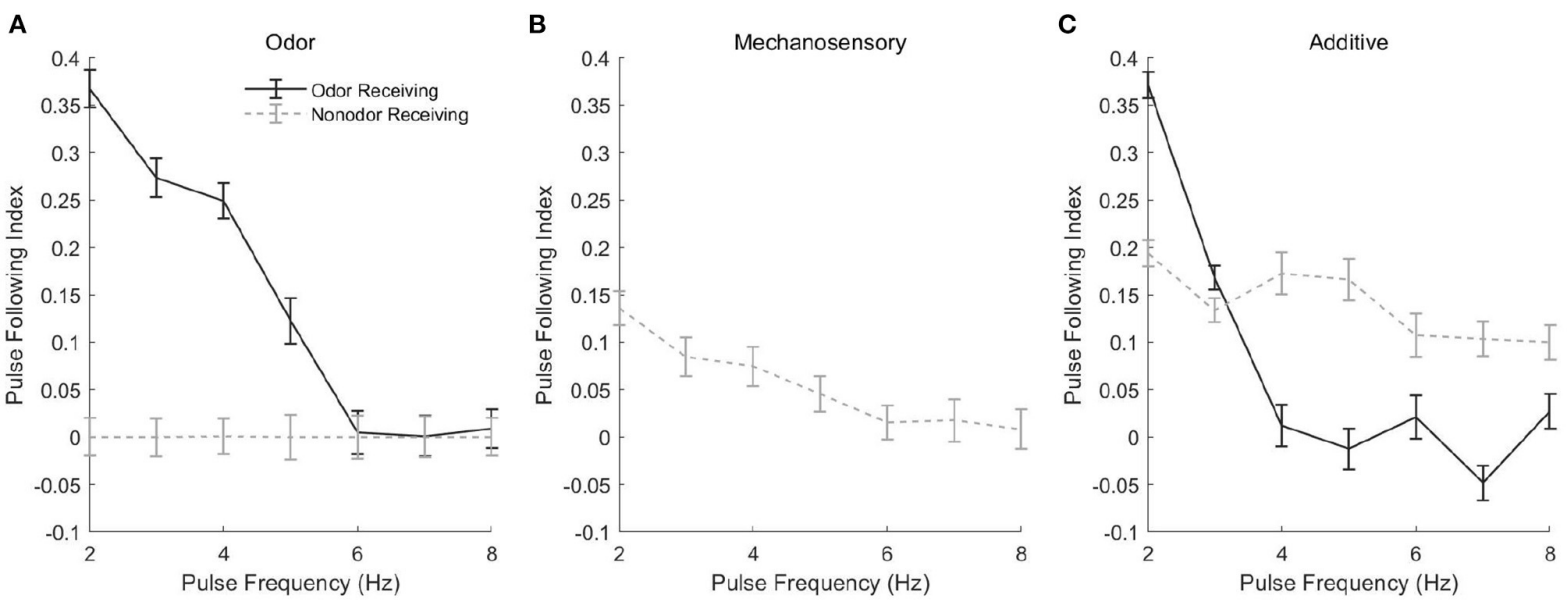
Pulse Following Index as a function of pulse frequency for a single odor-receiving and a single non odor-receiving PN from the network, in the odor only **(A)**, mechanosensory only **(B)**, and additive sensory integration **(C)** stimulus scenarios. The pulse following index is a measure of the ability of the PN response to track the temporal dynamics of pulsatile stimulus delivery, with values above zero signifying sensitivity to pulsatile dynamics and a value of zero implying a lack of discernment of pulse structure (see section 4 for details). Data are averaged over 50 trials.

Spike rasters from our model in the case of a 3 Hz (Figure 5, left panels) or 7 Hz (Figure 5, right panels) stimulus pulse train show similar behavior, with PNs tracking pulses via bursts or exhibiting tonic firing throughout the pulse train, depending on pulse frequency. In the odor only stimulus case (Figure 5A), PNs which receive odor stimulation appear capable of tracking a 3 Hz pulse train but not a 7 Hz pulse train (unstimulated PNs are quiescent), while in the mechanosensory only stimulus case (Figure 5B), PNs appear capable of tracking pulses at both 3 and 7 Hz. This is quantified in Figure 6, in which we employ a metric that we term the “pulse following index,” where values above zero signify an ability of the PN response to track pulse frequency and a value of zero implying an unwavering firing rate (i.e., a lack of ability to track pulse frequency). As suggested by the spike rasters in Figures 5A,B, the pulse following index (as a function of pulse frequency) declines to zero at a lower cutoff frequency in the odor only stimulus scenario (Figure 6A) than in the mechanosensory only stimulus scenario (Figure 6B). These observations are due to the brief nature of PN responses to isolated mechanosensory input in comparison to isolated odor input, arising as a consequence of global network dynamics that are discussed in detail in prior work (Tuckman et al., 2021); the relative brevity of PN responses to mechanosensory input allow these responses to track higher pulse frequencies than responses to odor input.

Interestingly, in the additive sensory integration stimulus scenario (Figure 5C), PNs which receive both odor and mechanosensory stimulation appear to have a cutoff frequency between 3 and 7 Hz, while PNs that receive only mechanosensory stimulation appear capable of tracking pulses at either 3 or 7 Hz (i.e., have a cutoff frequency >7 Hz). Figure 6C quantifies this difference, showing that the pulse following index (as a function of pulse frequency) declines to zero at a relatively low cutoff frequency (~4 Hz) for PNs within odor-receiving glomeruli, while remaining positive for pulse frequencies up to at least 8 Hz for PNs within non odor-receiving glomeruli. The large level of excitation to PNs within odor-receiving glomeruli (odor plus mechanosensory input) leads to relatively lengthy responses that slow inhibition from LNs cannot rapidly curtail, while the relatively weak excitation to PNs within non odor-receiving glomeruli (mechanosensory input alone) leads to rapid truncation of these PN responses by global slow inhibition shortly following pulse onset, and hence the latter set of PNs are able to track pulses at higher frequencies. Thus, we emphasize that, in the additive scenario, it appears that PNs that receive both odor and mechanosensory stimulation are adept at tracking pulse length (see previous section) but poor at tracking pulse frequency, while PNs that receive mechanosensory stimulation alone perform poorly in tracking pulse length (see previous section) but are effective at tracking pulse frequency, suggesting a possible division of roles between odor-receiving glomeruli and non odor-receiving glomeruli within the AL during high wind speed odor tracking.

#### 2.2.2. Conductance Dependence of Frequency Tracking

**Figure 7:**
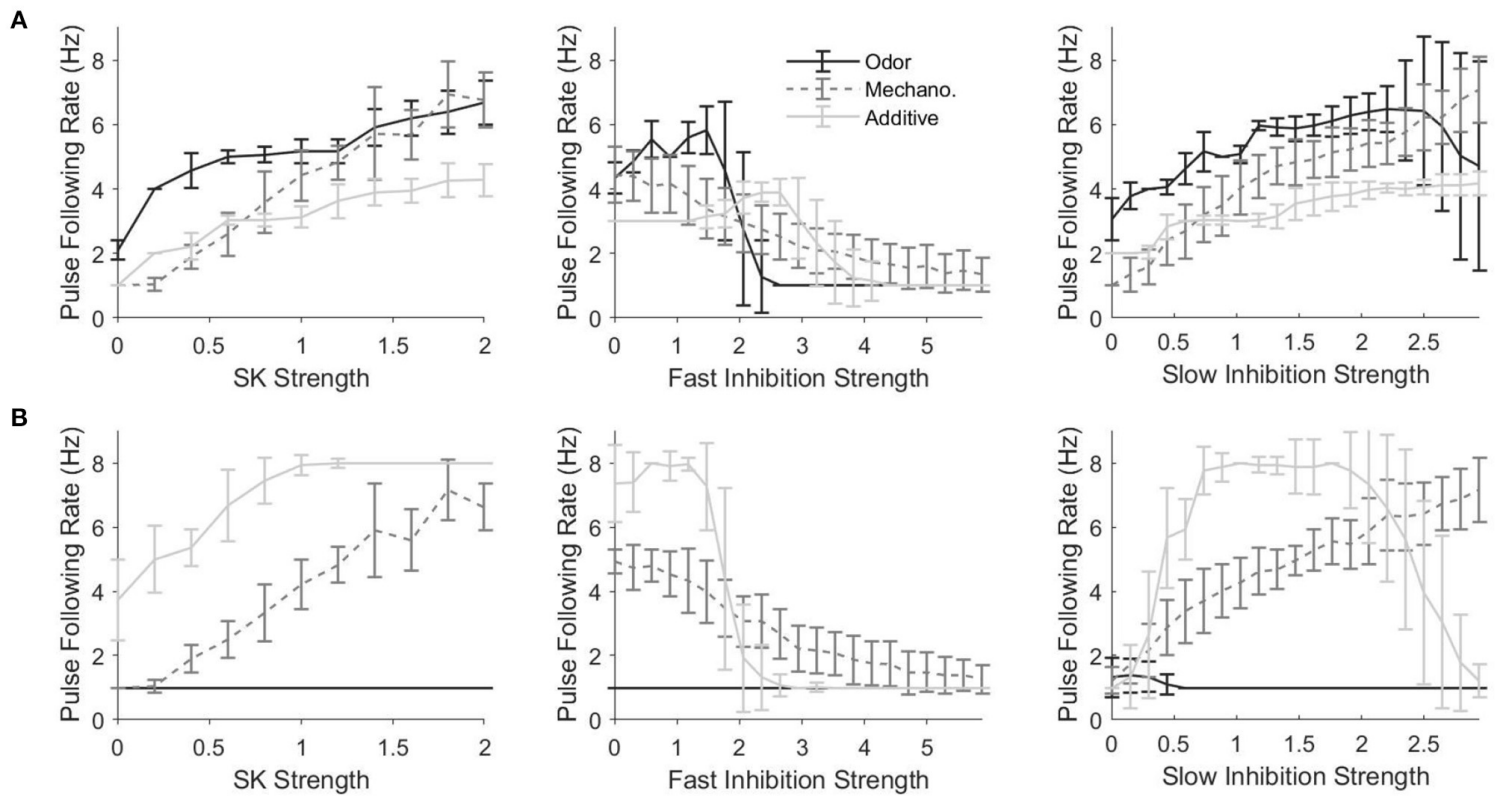
Pulse Following Rate as a function of the strength of the SK current (left), fast inhibitory synapses (center), and slow inhibitory synapses (right) for a single odor-receiving **(A)** and a single non odor-receiving **(B)** PN within the network. Data are shown for the odor only, mechanosensory only, and additive sensory integration stimulus scenarios. The pulse following rate is a measure akin to a cutoff frequency—i.e., the highest pulse frequency at which the PN response can track the temporal structure of stimuli delivered in the form of a train of pulses (see section 4 for details). Values on the x-axis are normalized by the standard values of the SK current, fast inhibitory synapses, or slow inhibitory synapses within the network, with a value of 1 indicating standard strengths. In the standard network, SK current strengths vary from PN to PN, and are drawn from a Gaussian distribution; in the simulations in this figure, SK current strengths are fixed across PNs, and the standard value of the SK current strength is taken as the mean of Gaussian employed in the standard network. Data are averaged over 50 trials.

In order to assess the effect of various components on the ability of PNs to track the temporal dynamics of stimulus pulses, we develop a metric, akin to a cutoff frequency (i.e., the highest pulse frequency that a PN is capable of tracking), that we term the “pulse following rate.” Figure 7 shows the pulse following rate as a function of the strength of different network currents for a PN in an odor-receiving glomerulus (Figure 7A) or non odor-receiving glomerulus (Figure 7B). For a PN in an odor-receving glomerulus, for all three stimulus scenarios, tracking of pulse frequency tends to improve with the strength of the SK current (Figure 7A, left) or slow inhibitory synapses (Figure 7A, right), but declines as fast inhibitory synapses are strengthened (Figure 7A, center). The SK current is an auto-inhibitory intrinsic current that accumulates with PN spiking and serves to self-curb further firing activity, while slow inhibition is a synaptic current which rises and accumulates gradually; both of these network components tend to permit PN spiking at the inception of a stimulus pulse with their inhibitory effects strengthening *after* the onset of PN spiking, and hence increasing the potency of these currents tends to yield briefer PN responses at pulse onset with more rapid truncation of PN responses following pulse onset, entailing a higher pulse following rate. Fast inhibition, on the other hand, rises and equilibrates rapidly (i.e., supplies an approximately constant mean level of inhibition to PNs throughout a pulse and does *not* tend to preferentially suppress the tail end of PN responses); thus, increasing the potency of fast inhibition does not tend to enhance pulse following rate, with very large fast inhibitory synaptic strengths tending to suppress PN responses altogether (yielding very low pulse following rates).

As discussed above, in the additive stimulus scenario, PNs that receive only mechanosensory input appear to be better at tracking pulse frequency than PNs that receive both odor and mechanosensory stimulation, an observation which is borne out by the fact that pulse following rates in general (for the additive case) are higher for a PN in a non odor-receiving (Figure 7B) vs. odor-receiving (Figure 7A) glomerulus. Furthermore, for a PN within a non odor-receiving glomerulus in the additive stimulus case, pulse following rate tends to increase with the strength of the SK current (Figure 7B, left) or slow inhibitory synapses (Figure 7B, right), while declining for high potency fast inhibitory synapses (Figure 7A, center), which occurs for similar reasons as above for the case of a PN within an odor-receiving glomerulus (Figure 7A). However, for very large slow inhibition strength values the pulse following rate tends to dip for a PN in a non odor receiving glomerulus in the additive stimulus case; this occurs because, for these very large values, the large level of excitation to the network as a whole (both mechanosensory and odor input) leads to vigorous global LN activation, and this vigorous activity, combined with the high potency of slow inhibitory synapses, yields a sufficient inhibitory drive to PNs within non odor-receiving glomeruli (which receive weak stimulation from mechanosensory input alone) to suppress responses at pulse onset even with the gradual rise time of the slow inhibitory current (hence impairing PN ability to track pulse frequency).

#### 2.2.3. Effect of Odor Tuning on Frequency Tracking

**Figure 8:**
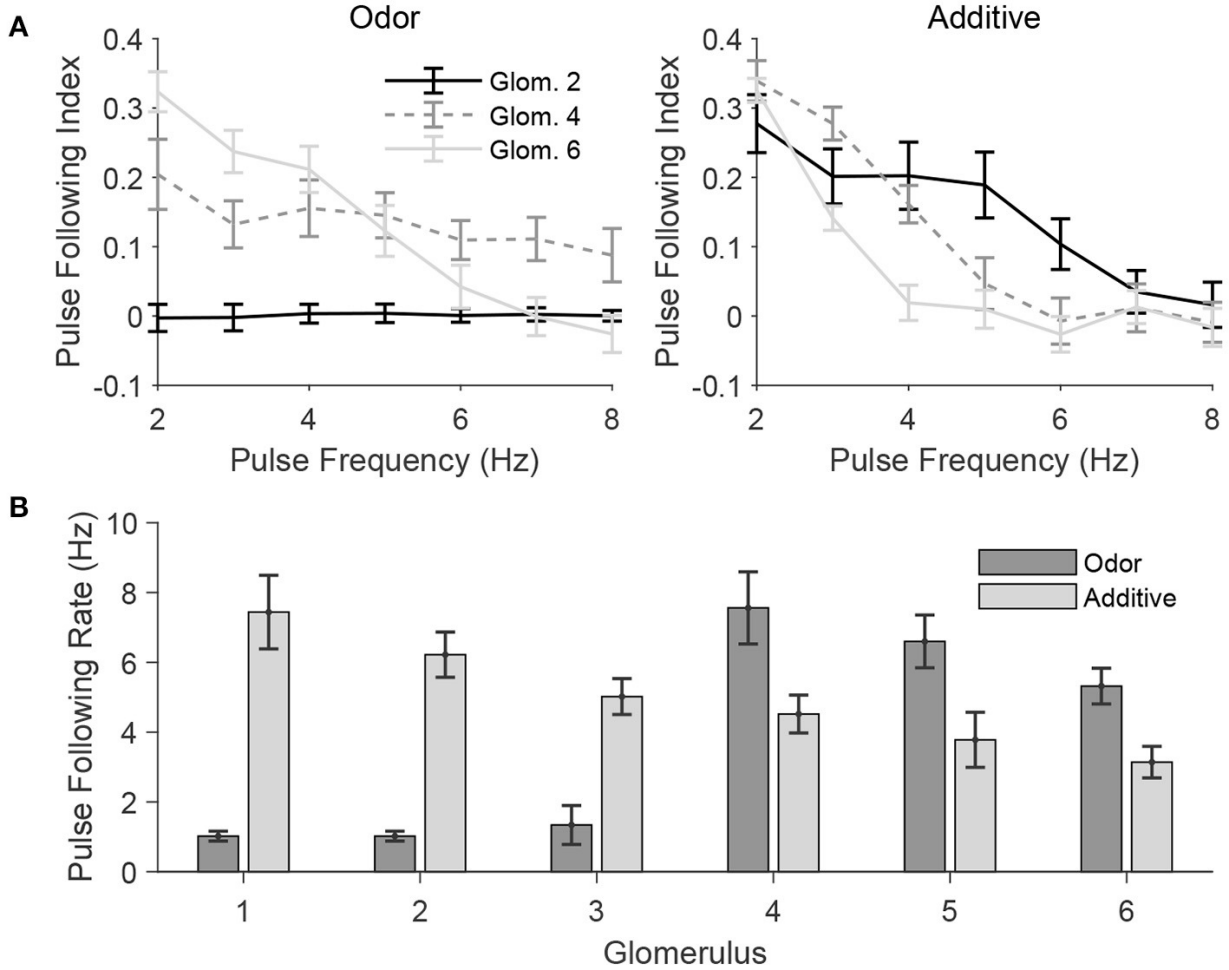
Pulse frequency tracking in the presence of varied odor tuning across glomeruli. In these simulations, odor is simulated by delivering odor-induced stimulus current to glomeruli 1, 2, 3, 4, 5, and 6 at 0, 0.2, 0.4, 0.6, 0.8, and 1, respectively, times the strength of the odor-induced stimulus current in the standard network (for those glomeruli in the standard network that are stimulated by odor). The total level of odor-induced excitation to the entire network is similar to that in the standard network, except with excitation delivered in a graded manner across glomeruli rather than three glomeruli receiving fixed positive excitation and the other three glomeruli receiving no excitation. **(A)** Pulse following index of net PN activity within a glomerulus plotted as a function of pulse frequency for several glomeruli, in the absence (left; Odor) or presence (right; Additive) of mechanosensory input. **(B)** Pulse following rate of net PN activity within a glomerulus for each glomerulus in the network, in the absence (Odor) or presence (Additive) of mechanosensory input. Data are averaged over 50 trials.

In a natural setting, a given odor likely activates some glomeruli more extensively than others, producing odor tuning profiles for each glomerulus. In order to further quantify the effect of odor and mechanosensory stimulation on the ability of PNs to track the temporal dynamics of pulsatile stimuli, in Figure 8 we employ a stimulus paradigm in which odor input is sent to all glomeruli, but in a graded fashion—glomerulus 1 receives no odor input, while the magnitude of odor input is progressively increased from glomerulus 2 to glomerulus 6, with mechanosensory input either absent (odor only case) or sent equally to all glomeruli in standard fashion (additive case). The gradation of odor input is performed in a manner to ensure that the net odor-induced level of excitation to the model AL is the same as in the standard odor stimulation paradigm employed previously (in which only glomeruli 1, 2, and 3 receive odor input, with odor input to the three glomeruli being equal in amplitude). Thus, the paradigm employed in Figure 8 allows assessment of the effect of varying levels of odor input (or, equivalently, variation across glomeruli in tuning for a given odor) on the ability of PNs to track pulse frequency, in either the absence or presence of mechanosensory input, while maintaining the same net level of excitation to the AL as in the odor only and additive stimulus scenarios, respectively, employed above.

In the odor only case (Figure 8A, left), the pulse following index remains high for higher pulse frequencies for a PN in a glomerulus that receives a moderate amount of odor input (glomerulus 4), while remaining near zero or dropping to zero at higher pulse frequencies for PNs within glomeruli that receive little odor input (glomerulus 2) or a large level of odor input (glomerulus 6), respectively; in the absence of mechanosensory input, a moderate level of odor input is ideal for tracking pulse frequency—excitation is sufficiently strong to allow a brief burst of PN spiking at pulse onset, but weak enough to allow slow inhibition and the SK current to rapidly suppress PN spiking shortly following the initial burst. In the additive case (Figure 8A, right), the pulse following index remains high for higher pulse frequencies for the PN receiving the least amount of odor input (glomerulus 2) and declines more rapidly with pulse frequency as odor input is increased for PNs in glomeruli 4 and 6; since all network PNs receive a baseline level mechanosensory input in this stimulus case, those PNs which are sent the least amount of odor input receive the moderate level of net excitation necessary to effectively track pulse frequency. Accordingly, Figure 8B shows that for the odor only case, pulse following rate is highest for glomerulus 4, while for the additive case, pulse following rate declines steadily from glomerulus 1 to glomerulus 6.

## 3. Discussion

In this work, we study a model of the moth AL incorporating both mechanosensory input, as a relatively weak but more broadly distributed signal, as well as olfactory input, as a relatively strong signal but with focal glomerular targeting, and we assess the effects of mechanosensory input, along with various components, on the ability of PN activity to track the temporal length of a stimulus pulse or the temporal dynamics of a train of stimulus pulses. Notably, we find that when sensory integration of both stimulus modalities is taken into account, PNs receiving both olfactory and mechanosensory input are adept at tracking the temporal length of a single stimulus pulse but are inefficient at tracking the temporal dynamics of a train of stimulus pulses (i.e., at tracking pulse frequency), while PNs receiving mechanosesory input alone (and no odor input) are unable to monitor the length of a single stimulus pulse but are proficient at tracking pulse frequency during a train of stimulus pulses. We therefore hypothesize that the ratio of these two kinds of input plays a large role in determining if a PN mainly encodes pulse duration or pulse frequency. Furthermore, we find, in general, that the ability of PNs to monitor pulse length is enhanced when the strength of the intrinsic SK current or synaptic slow inhibition is relatively low, while the efficacy of pulse frequency tracking is augmented by relatively high strength values of the SK current or slow inhibition.

In particular, this suggests an intriguing connection to the variable phase II spiking patterns of PNs. Within the moth AL, shortly after stimulus onset and following a brief initial hyperpolarization, stimulus-induced spiking ensues (phase II of the response). Experimental data shows that during phase II, spike patterns can vary broadly across active PNs; some PNs spike in sporadic bursts, while others fire more continuously, with a range of spike patterns between the two extremes. Moreover, the phase II dynamics exhibited by a PN show no spatial dependency—in fact, a significant amount of intraglomerular variability has been observed during stimulation (Christensen et al., 2000; Lei et al., 2011). In prior work with the current model (Tuckman et al., 2021), we show that, within our model, phase II spiking variability arises as a consequence of the SK current—lower SK current strength tends to yield more continuous phase II activity, while higher SK current strength results in phase II activity consisting more of sporadic bursts. The above results suggest the tantalizing possibility that phase II spiking variability may be related to the role that a PN plays in pulse tracking—i.e., a low SK strength value entails continuous phase II firing and effective tracking of pulse length (but not pulse frequency), while a high SK strength value entails burst-like phase II firing and effective tracking of pulse frequency (but not pulse length).

Moreover, these effects of the SK current on phase II activity suggested by our model are supported by pharmacological experiments. Application of bicuculline methiodide onto the AL of the male moth *M. sexta* leads to pheromone-responsive PNs prolonging their phase II response well beyond the duration of a pheromone stimulus (Lei et al., 2009). Interestingly, one of the effects of bicuculline methiodide is believed to be blockage of SK channels (Khawaled et al., 1999). Additionally, another modeling study where SK channels were blocked also showed prolonged excitatory responses in PNs (Belmabrouk et al., 2011).

### 3.1. Functional Division of Roles Within the AL

The results of the present work lead to an interesting hypothesis pertaining to a possible division of roles within the moth AL, a division in an odor-specific functional sense rather a strict anatomic sense. Upon encountering an environmental odor, a focal subset of AL glomeruli receives odor-induced input, while a broader (or at least disparate, but likely overlapping) set of glomeruli receives mechanosensory input due to wind stimulation. PNs within glomeruli strongly activated by the odor itself, then, tend to predominantly encode odor features such as odor identity and concentration (as well as tracking the temporal length of a prolonged odor encounter), while PNs that receive predominantly mechanosensory input tend to be more effective at tracking the temporal dynamics of brief odor pulses encountered in rapid fashion. Thus, the functional role of the various AL glomeruli may shift based on the specific odor that is being sensed, and depend on the specific glomeruli activated by the odor—those glomeruli strongly activated by the odor tend to encode odor features, while those glomeruli not activated or activated less by the odor, but stimulated by mechanosensory input, tend to track the temporal dynamics of odor pulses.

There is a conceivable advantage arising from such functional division of glomerular roles. The dual strategies allow animals to react quickly to track an odor plume as well as evaluate odor quality during navigation. For example, some phylogenetically-linked insect species are distributed in a common habitat, with the females of these closely-related species releasing sex pheromones that are composed of similar compounds. In a competitive situation, males must launch their plume-tracking program swiftly upon detection of a minute trace of pheromones (emanating from a distant source) in order to increase the probability of successful mating. However, it is beneficial for the male to evaluate the correctness of the pheromone stimulus—i.e., to determine that the pheromone is from a female of its own species and not a closely related separate species—before completing an energetically costly journey. Mid-flight, non pheromone-receiving glomeruli may function to register contact with high wind speed stimulus pulses (which are likely to efficaciously deliver odor to antennal receptors) and engage flight behavior to intercept odor filaments, whereas pheromone-receiving glomeruli may serve to extract odor quality information via more prolonged (but less temporally precise) responses. This information may be used to direct the animal to continue tracking until reaching the target, if the odor is determined to be the right type of pheromone, or to abort tracking if the pheromone is determined to be from a different species. Behavioral experiments support this hypothetical scenario. In a wind tunnel experiment, male *M. sexta* moths were subjected to stimulation by female sex pheromones, in which pheromone mixtures, made of the same two compounds but in different ratios, were used. The male moths initiated odor-tracking flight behavior regardless of which pheromone mixtures were placed upwind, but only hovered in front of and landed on the septum emitting pheromone compounds in the natural ratio for the species (Martin et al., 2013). This observation suggests that the pheromone plume was not only tracked but also evaluated by the male moths.

Additionally, such a picture of an odor-dependent functional division of roles among AL glomeruli could also potentially explain *intraglomerular* variability in phase II PN response patterns (Christensen et al., 2000; Lei et al., 2011). Within a glomerulus, different PNs may exhibit varying strengths of the intrinsic SK current; PNs within a glomerulus that have high SK current strength tend to exhibit burst-like phase II behavior while those that have lower SK strength values tend to exhibit more continuous phase II firing (Tuckman et al., 2021), and hence PNs within a glomerulus that display burst-like phase II response patterns may be specifically specialized to track the temporal dynamics of pulsatile odor encounters, while those that exhibit continuous phase II spiking may be specialized to track odor features or the length of prolonged odor pulses. If an odor-dependent functional division of roles is indeed present within the moth AL, then such intraglomerular PN variability is a necessary feature—since the subset of glomeruli that encode odor features and the disparate subset that tend to track pulse dynamics *change* from one odor to the next, this implies that each individual glomerulus must be capable of playing both roles, and therefore must contain *both* types of PN specializations. Furthermore, it is interesting to speculate that, in accordance with intraglomerular specialization of PNs, it is possible that those PNs within a glomerulus specialized for tracking pulse dynamics might tend to receive a higher ratio of mechanosensory to odor input than those PNs specialized to encode odor features (and pulse length), though empirical studies have not yet been conducted to evaluate such a proposition.

### 3.2. Dynamic Modulation of Pulse Tracking

The role of mechanosensory input in facilitating the tracking of the temporal structure of pulsatile odor encounters suggests the possibility of a dynamic modulation of the AL network state, with the AL operating in distinct regimes depending on behavioral necessity. The presence of significant mechanosensory input may induce the entire AL network, as a whole, to shift into a regime that is more poised to track the temporal dynamics of rapid and brief odor pulses and less geared toward fine odor discrimination, while minimal mechanosensory input may place the AL network in a global regime that is more suited to fine odor discrimination (presumably enhanced by lengthier PN responses) and less adept at tracking pulse dynamics. Such regimes may roughly correspond to the behavioral state of the insect—when flying and seeking an odor source, the ensuing high level of mechansensory input and lower level of olfactory input (due to distance from the odor source) may create a general global tendency to shift PNs toward being better trackers of rapid pulse dynamics, while when sitting relatively still near an odor source, a lower level of mechanosensory input and higher olfactory input (due to proximity to the odor source) may shift PNs toward exhibiting lengthier responses and being better olfactory discriminators.

## 4. Methods

We construct a spiking model of the AL network that strives to attain enough architectural complexity to achieve the complex dynamics of the AL while maintaining enough simplicity to allow for investigation of core mechanistic components. Below, we elaborate the components and connectivity of our model as well as the details of our analyses of model dynamics. Details are similar to those in our prior work with this model (Tuckman et al., 2021).

### 4.1. The Neuron Model

The model is composed of two subclasses of neurons: excitatory, cholinergic PNs, and inhibitory, GABAergic LNs. The membrane potential of the *jth* PN [$V_{PN}^{j}\left(t\right)$] or the *jth* LN [$V_{LN}^{j}\left(t\right)$] are modeled using integrate-and-fire dynamics by the following set of ODEs, which include both intrinsic and synaptic currents:


$$\begin{array}{l} \frac{d}{dt}V_{PN}^{j}\left(t\right)=-\frac{1}{\tau_{V}}\left(V_{PN}^{j}-V_{L}\right)-g_{SK}^{j}\left(t\right)\left(V_{PN}^{j}-V_{SK}\right)\\ \;-g_{stim}^{j}\left(t\right)\left(V_{PN}^{j}-V_{stim}\right)\\ \;-g_{exc}^{j}\left(t\right)\left(V_{PN}^{j}-V_{exc}\right)\\ \;-g_{inh}^{j}\left(t\right)\left(V_{PN}^{j}-V_{inh}\right)\\ \;-g_{slow}^{j}\left(t\right)\left(V_{PN}^{j}-V_{inh}\right) \end{array}$$

$$\begin{array}{l} \frac{d}{dt}V_{LN}^{j}\left(t\right)=-\frac{1}{\tau_{V}}\left(V_{LN}^{j}-V_{L}\right)-g_{stim}^{j}\left(t\right)\left(V_{LN}^{j}-V_{stim}\right)\\ \;-g_{exc}^{j}\left(t\right)\left(V_{LN}^{j}-V_{exc}\right)\\ \;-g_{inh}^{j}\left(t\right)\left(V_{LN}^{j}-V_{inh}\right)\\ \;-g_{slow}^{j}\left(t\right)\left(V_{LN}^{j}-V_{inh}\right) \end{array}$$

PN *j* in the model is equipped with an intrinsic slow potassium current (SK), and receives stimulus-induced input (from external background, odor, and mechanosensory sources), fast excitatory input from other PNs, fast inhibitory input from LNs, and slow inhibitory input from LNs. LN *j* in the model receives stimulus-induced input (from external background, odor, and mechanosensory sources), fast excitatory input from PNs, fast inhibitory input from other LNs, and slow inhibitory input from other LNs. In these equations, *V*_*L*_ = 0, $V_{exc}=V_{stim}=\frac{14}{3}$, and $V_{SK}=V_{inh}=-\frac{2}{3}$ (expressed in non-dimensional units) represent reversal potentials associated with leakage, excitation, and inhibition, respectively. The leakage timescale is given by τ_*V*_ = 20ms. Upon any neuron reaching a threshold voltage of *V*_*thres*_ = 1, a spike is recorded and its voltage subsequently reset to *V*_*L*_ = 0 (and held at *V*_*L*_ = 0 for a refractory period of τ_*ref*_ = 2 ms). The neuron model is based on a reduced dimensional integrate-and-fire model previously developed in the literature (Tao et al., 2004; Lei et al., 2016).

The term $g_{exc}^{j}\left(t\right)$ represents the membrane conductance of neuron *j* to excitatory synaptic input from PNs, and is modeled as follows:


$$g_{exc}^{j}\left(t\right)=\underset{s\in S}{\sum }S_{PN}\alpha_{exc}\left(t|s\right),\text{ where }\alpha_{exc}\left(t|s\right)=\frac{H\left(t-s\right)}{\tau_{exc}}e^{-\frac{\left(t-s\right)}{\tau_{exc}}}$$

In this equation, *S* represents the set of all spike times of all PNs presynaptic to neuron *j*. *S*_*PN*_ is the coupling strength of a network PN to neuron *j*; *S*_*PN*_ = 0.01 if neuron *j* is a PN, while *S*_*PN*_ = 0.006 if neuron *j* is an LN. α_*exc*_(*t*|*s*) is a function with instantaneous rise time and exponential decay time, with time constant τ_*exc*_ = 2 ms (whether neuron *j* is an LN or a PN).

The other synaptic conductances, $g_{inh}^{j}\left(t\right)$ and $g_{slow}^{j}\left(t\right)$, as well as the stimulus conductance, $g_{stim}^{j}\left(t\right)$, are modeled similarly:


$$g_{inh}^{j}\left(t\right)=\underset{s\in S}{\sum }S_{inh}\alpha_{inh}\left(t|s\right),\text{ where }\alpha_{inh}\left(t|s\right)=\frac{H\left(t-s\right)}{\tau_{inh}}e^{-\frac{\left(t-s\right)}{\tau_{inh}}}$$

$$g_{slow}^{j}\left(t\right)=\underset{s\in S}{\sum }S_{slow}\alpha_{slow}\left(t|s\right),\text{ where }\alpha_{slow}\left(t|s\right)=\frac{H\left(t-s\right)}{\tau_{slow}}e^{-\frac{\left(t-s\right)}{\tau_{slow}}}$$

$$g_{stim}^{j}\left(t\right)=\underset{s\in S}{\sum }S_{stim}\alpha_{stim}\left(t|s\right),\text{ where }\alpha_{stim}\left(t|s\right)=\frac{H\left(t-s\right)}{\tau_{stim}}e^{-\frac{\left(t-s\right)}{\tau_{stim}}}$$

For the $g_{inh}^{j}\left(t\right)$ and $g_{slow}^{j}\left(t\right)$ equations, *S* represents the set of all spike times of all LNs presynaptic to neuron *j*. For the $g_{stim}^{j}\left(t\right)$ equation, *S* represents the set of all spike times of the external input delivered to neuron *j*; these stimulus-induced spike times arise from simulation of background input, odor input, and mechanosensory input as Poisson processes of incoming spike events (see section 4.3 below for details). If neuron *j* is a PN, the coupling strengths are given by *S*_*inh*_ = 0.0169, *S*_*slow*_ = 0.0338, and *S*_*stim*_ = 0.004, while if neuron *j* is an LN, the coupling strengths are given by *S*_*inh*_ = 0.015, *S*_*slow*_ = 0.04, and *S*_*stim*_ = 0.0031. The fast inhibition and stimulus timescales are comparable to excitation, with τ_*inh*_ = τ_*stim*_ = 2 ms, while the slow inhibition time scale is dramatically slower, with τ_*slow*_ = 750 ms (whether neuron *j* is a PN or an LN).

Finally, the SK current is an intrinsic slow potassium current, displayed by only PNs, that activates upon spiking and serves to curb further spiking activity. Rather than an instantaneous jump, the rise time of the SK current is modeled as sigmoidal; this non-instantaneous rise time allows PNs to potentially emit multiple spikes prior to suppression of firing activity by the SK current. The SK current for PN *j* is modeled as follows:


$$g_{SK}^{j}\left(t\right)=\underset{s\in S}{\sum }S_{SK}\beta \left(t|s\right)$$

$$\beta \left(t|s\right)=\{\begin{array}{c} \frac{H\left(t-s\right)}{\tau_{SK}}\frac{e^{\frac{5\left(\left(t-s\right)-\tau_{rise}\right)}{\tau_{rise}}}}{1+e^{\frac{5\left(\left(t-s\right)-\tau_{rise}\right)}{\tau_{rise}}}},t\leq s+2\tau_{rise}\\ \frac{1}{\tau_{SK}}e^{\frac{-\left(t-\left(s+2\tau_{rise}\right)\right)}{\tau_{SK}}},t>s+2\tau_{rise} \end{array}$$

*S* represents the set of all firing times of PN *j*. The strength *S*_*SK*_ of the SK current is a randomly determined, but fixed, parameter, and hence varies from PN to PN; the value of *S*_*SK*_ for PN *j* is drawn from a normal distribution with mean μ = 0.5 and standard deviation σ = 0.2. While rare, it is possible for *S*_*SK*_ to be negative with this distribution, so any negative value for *S*_*SK*_ is manually set to 0. τ_*SK*_ = 250 ms, and the rise of the SK current is modeled as sigmoidal with a half-rise time of τ_*rise*_ = 25 ms. A distribution of SK current strengths across PNs ensures variability across PNs in phase II firing behavior.

In the above, *H*(*t*) is the standard Heaviside Step Function:


$$H\left(t\right)=\{\begin{array}{c} 1,t\geq 0\\ 0,t<0 \end{array}$$

### 4.2. Network Architecture

Our AL model consists of six glomeruli, with each glomerulus consisting of 10 PNs and 6 LNs; connectivity within glomeruli is dense in comparison with relatively sparse connectivity across glomeruli. Synaptic connections within the model are randomly determined but fixed, with the probability of a synaptic connection varying within and across glomeruli and dependent on cell type. Within a glomerulus, the PN → PN, PN → LN, LN → PN, LN → LN connection probabilities are given by 0.75, 0.75, 0.38, 0.25, respectively. Long-range connections (i.e., connections across glomeruli) are mediated exclusively by LN → PN synapses, and the cross-glomerular LN → PN connection probability is given by 0.38.

It is worth mentioning that the model itself is quite robust, with the exact input parameters provided not essential to producing reasonable behavior. Rather, we find that combinations of parameters, and hence the relative strength of disparate network components, are important for producing realistic behavior. For example, we find that slow inhibition must be sufficiently strong, relative to stimulus-induced inputs, to suppress PN spiking if the global LN network is activated, yet not so strong as to silence PN activity upon only focal activation of a few glomeruli. Likewise, we find that the strength of the SK current must fall within a broad range of values, relative to the strength of stimulus-induced inputs and LN inhibition, with the lower end of this range yielding homogeneous PN spiking activity and the higher end of this range yielding burst-like PN behavior. Hence, our parameter choices represent a single point drawn from a relatively large cloud (in multidimensional parameter space) of parameter combinations that produce physiologically reasonable behavior.

### 4.3. Stimulus Modeling

Rather than explicitly modeling the behavior of ORNs or the cells responsible for mechanosensory sensory inputs, input to each cell within the network is modeled as a Poisson process of incoming spikes. An incoming spike to neuron *j* within the network is modeled as an instantaneous jump in $g_{stim}^{j}\left(t\right)$ of size 0.004 if neuron *j* is a PN, or 0.0031 if neuron *j* is an LN, followed by exponential decay with time constant τ = 2 ms (whether neuron *j* is a PN or an LN). Each cell has three potential sources of input; all cells receive a background rate of λ_*back*_ = 3.6 spikes/ms, while odor input (simulating the presence of a single odor) is delivered at a maximum rate of $\lambda_{odor}^{max}=3.6$ spikes/ms and mechanosensory input is delivered at a maximum rate of $\lambda_{mech}^{max}=1.8$ spikes/ms. The total rate of incoming spikes for the *jth* cell is therefore given by:


$$\begin{array}{l} \lambda_{tot}^{j}\left(t\right)=\lambda_{back}\\ \;+\underset{t_{on}\in \Gamma_{t}}{\sum }\left(\lambda_{odor}^{j}O^{j}\left(t;t_{on},t_{off}\right)+\lambda_{mech}^{j}M^{j}\left(t;t_{on},t_{off}\right)\right), \end{array}$$

where *O*^*j*^(*t*) and *M*^*j*^(*t*) are functions that range between 0 and 1 and serve to model the temporal dynamics of odor and mechanosensory input pulses, respectively. Γ_*t*_ is the set of all pulse start times occurring prior to time *t*. The *t*_*on*_ and *t*_*off*_ parameters in the *O*^*j*^ and *M*^*j*^ functions indicate the start and end times of pulses, respectively. For simulations where a train of pulses is presented, pulses are 50 ms in duration (*t*_*off*_−*t*_*on*_ = 50).

To simulate background AL activity, we set $\lambda_{odor}^{j}=0$ and $\lambda_{mech}^{j}=0$ for all *j*. To simulate an odor pulse (without simulation of mechanosensory input) presented at time *t*_*on*_ and removed at time *t*_*off*_, we send odor-induced input to all cells within three out of six model glomeruli (with the glomerular subset signifying odor identity); we therefore set $\lambda_{odor}^{j}=0$ if cell *j* belongs to an unstimulated glomerulus and $\lambda_{odor}^{j}=\lambda_{odor}^{max}$ if cell *j* belongs to a stimulated glomerulus, and set $\lambda_{mech}^{j}=0$ for all *j*. To simulate a pulse of mechanosensory input (without an accompanying olfactory stimulus) from time *t*_*on*_ to time *t*_*off*_, we set $\lambda_{odor}^{j}=0$ and $\lambda_{mech}^{j}=\lambda_{mech}^{max}$ for all *j*. Hence, within the model, olfactory input stimulates a focal glomerular subset, while mechanosensory input represents a global signal delivered to the entirety of the AL.

In addition to simulating olfactory and mechanosensory input in isolation, we also simulate the two in combination. To simulate a stimulus pulse (from time *t*_*on*_ to time *t*_*off*_) consisting of a single odor accompanied by a mechanosensory signal, we employ the additive sensory integration paradigm. In the additive sensory integration paradigm, we set $\lambda_{odor}^{j}=0$ if cell *j* belongs to a glomerulus not responsive to the odor and $\lambda_{odor}^{j}=\lambda_{odor}^{max}$ if cell *j* belongs to a glomerulus activated by the odor, and set $\lambda_{mech}^{j}=\lambda_{mech}^{max}$ for all *j*. Hence, the additive paradigm simply “adds” together the isolated olfactory and mechanosensory signals.

The function *O*^*j*^(*t*) represents the temporal dynamics of the olfactory component of a stimulus pulse beginning at time *t*_*on*_ and ending at time *t*_*off*_. *O*^*j*^(*t*) = 0 for *t* < *t*_*on*_; at time *t*_*on*_, *O*^*j*^(*t*) increases from 0 to 1 with a prescribed rise time, while for *t*>*t*_*off*_, *O*^*j*^(*t*) decreases from 1 to 0 with a prescribed decay time. If neuron *j* is a PN, rise is sigmoidal with a half-rise time of τ_*rise*_ = 35ms, while decay is exponential with τ_*decay*_ = 384ms:


$$\text{If }j\text{ is a PN, }O^{j}\left(t;t_{on},t_{off}\right)=\{\begin{array}{l} H\left(t-t_{on}\right)\frac{e^{\frac{5\left(\left(t-t_{on}\right)-\tau_{rise}\right)}{\tau_{rise}}}}{1+e^{\frac{5\left(\left(t-t_{on}\right)-\tau_{rise}\right)}{\tau_{rise}}}},\\ \;t\leq t_{on}+2\tau_{rise}\\ 1,\\ \;t_{on}+2\tau_{rise}<t\leq t_{off}\\ e^{\frac{-\left(t-t_{off}\right)}{\tau_{decay}}},\\ \;t_{off}<t \end{array}$$

If neuron *j* is a LN, rise is instantaneous, while decay is exponential with τ_*decay*_ = 384 ms:


$$\text{If }j\text{ is an LN, }O^{j}\left(t;t_{on},t_{off}\right)=\{\begin{array}{l} H\left(t-t_{on}\right),\;t\leq t_{off}\\ e^{\frac{-\left(t-t_{off}\right)}{\tau_{decay}}},{\;t}_{off}<t \end{array}$$

Similarly, the function *M*^*j*^(*t*) represents the temporal dynamics of the mechanosensory component of a stimulus pulse beginning at time *t*_*on*_ and ending at time *t*_*off*_. *M*^*j*^(*t*) = 0 for *t*<*t*_*on*_; at time *t*_*on*_, *M*^*j*^(*t*) increases from 0 to 1 with a prescribed rise time, while for *t*>*t*_*off*_, *M*^*j*^(*t*) decreases from 1 to 0 with a prescribed decay time. If neuron *j* is a PN, rise is instantaneous and decay is exponential with τ_*decay*_ = 384 ms:


$$\text{If }j\text{ is a PN, }M^{j}\left(t;t_{on},t_{off}\right)=\{\begin{array}{l} H\left(t-t_{on}\right),\;t\leq t_{off}\\ e^{\frac{-\left(t-t_{off}\right)}{\tau_{decay}}},{\;t}_{off}<t \end{array}$$

If neuron *j* is a LN, τ_*rise*_ = 300ms and τ_*decay*_ = 384ms:


$$\text{ If}j\text{ is an LN, }M^{j}\left(t;t_{on},t_{off}\right)=\{\begin{array}{l} H\left(t-t_{on}\right)\frac{e^{\frac{5\left(\left(t-t_{on}\right)-\tau_{rise}\right)}{\tau_{rise}}}}{1+e^{\frac{5\left(\left(t-t_{on}\right)-\tau_{rise}\right)}{\tau_{rise}}}},\\ \;t\leq t_{on}+2\tau_{rise}\\ 1,\\ {\;t}_{on}+2\tau_{rise}<t\leq t_{off}\\ e^{\frac{-\left(t-t_{off}\right)}{\tau_{decay}}},\\ {\;t}_{off}<t \end{array}$$

We included a significantly longer rise time for mechanosensory input to LNs, relative to that for PNs, in order to ensure that global LN inhibition at the inception of a stimulus pulse (mediated by fast inhibitory synapses) is not overwhelmingly powerfully enough to prevent PN spiking altogether, and that substantial suppression of PN spiking must await the lengthy activation time of slow inhibitory synapses. This assumption, however, is not necessarily required to obtain physiologically reasonable dynamics – for example, weakening fast inhibitory synapses from LNs to PNs or strengthening slow inhibition while reducing the density of LN → PN synapses can yield similar dynamical effects without such a disparity in rise times. Since the dynamics of mechanosensory input to AL cells has not yet been studied within the experimental literature, we (somewhat arbitratrily, due to ignorance of the actual physiological mechanism at play) chose to include this mechanism of a disparity in rise times to ensure robust PN spiking at stimulus onset. However, we note that including a disparity in rise times does not affect the basic dynamical behavior of the model, other than delaying suppression of PN spiking at stimulus onset. We also note that in this stimulus modeling scheme, λ_*back*_ can be thought of as background input arising from mild stimulation due to ambient low wind speed, and λ_*mech*_ can be thought of as describing the additional mechanosensory input impinging upon cells via an encounter with a high wind speed pulse.

### 4.4. Simulation and Data Analysis

Means and standard deviations were taken over 50 trials. Best fit lines such as those found in Figures 3, 4 were determined via a least squares linear regression. The PN response lengths used in Figures 3, 4 were determined as the length of time between the first spike following initiation of stimulation and the end of the response. The response was considered ended at the moment an interspike interval within the induced spike train exceeded three times the average interspike interval of the first three initial spikes.

The pulse following index, as seen in Figures 6, 8, was determined by taking the difference in the autocorrelation of the spike raster starting at the inception of the first pulse and the autocorrelation at the end of the first pulse. This “peak minus trough” difference of the autocorrelation plot was the reported pulse following index. Based on the results of Figure 6, a pulse frequency index cutoff of 0.05 was used to determine the pulse following rates in Figures 7, 8. In other words, the pulse following rate is the maximum rate for which a PN's pulse following index is greater than or equal to 0.05. All autocorrelations were calculated using the built-in Matlab xcorr function.

Numerical simulations were carried out using the Euler Method with a time step of Δ*t* = 0.1 ms. Model code was written in C++ with data analysis and plotting carried out in Matlab. Model code is available at https://gitlab.com/HarrisonTuckmanWM/antennal-lobe-model-2-0.

## Data Availability Statement

Publicly available datasets were analyzed in this study. This data can be found at: https://gitlab.com/HarrisonTuckmanWM/antennal-lobe-model-2-0.

## Author Contributions

All authors listed have made a substantial, direct and intellectual contribution to the work, and approved it for publication.

## Funding

This research was partly funded by Award 2014217, a subaward to BHS as part of the NSF/CIHR/DFG/FRQ/UKRI-MRC Next Generation Networks for Neuroscience Program.

## Conflict of Interest

The authors declare that the research was conducted in the absence of any commercial or financial relationships that could be construed as a potential conflict of interest.

## Publisher's Note

All claims expressed in this article are solely those of the authors and do not necessarily represent those of their affiliated organizations, or those of the publisher, the editors and the reviewers. Any product that may be evaluated in this article, or claim that may be made by its manufacturer, is not guaranteed or endorsed by the publisher.
